# Chromatin states responsible for the regulation of differentially expressed genes under ^60^Co~γ ray radiation in rice

**DOI:** 10.1186/s12864-017-4172-x

**Published:** 2017-10-12

**Authors:** Xiucai Pan, Yuan Fang, Xueming Yang, Dongyang Zheng, Lifen Chen, Lei Wang, Jin Xiao, Xiu-e Wang, Kai Wang, Zhukuan Cheng, Hengxiu Yu, Wenli Zhang

**Affiliations:** 1 State Key Laboratory for Crop Genetics and Germplasm Enhancement, Nanjing Agriculture University, Nanjing, Jiangsu 210095 China; 2 Provincial Key Laboratory of Agrobiology, Institute of Biotechnology, Jiangsu Academy of Agricultural Sciences, Nanjing, 210014 China; 3 Center for Genomics and Biotechnology, Haixia Institute of Science and Technology (HIST), Fujian Agriculture and Forestry University, Fuzhou, Fujian 35002 China; 4 State Key Laboratory of Plant Genomics and Center for Plant Gene Research, Institute of Genetics and Developmental Biology, Chinese Academy of Sciences, Beijing, China; 5 Key Laboratory of Crop Genetics and Physiology of Jiangsu Province/Key Laboratory of Plant Functional Genomics of Ministry of Education, Yangzhou University, Yangzhou, China; 6 JiangSu Collaborative Innovation Center for Modern Crop Production (JCIC-MCP), Nanjing Agriculture University, Nanjing, Jiangsu 210095 China

**Keywords:** Gene expression, Histone modifications, Chromatin states, Ionizing radiation, *Oryza sativa*

## Abstract

**Background:**

The role of histone modifications in the DNA damage response has been extensively studied in non-plant systems, including mammals and yeast. However, there is a lack of detailed evidence showing how chromatin dynamics, either an individual mark or combined chromatin states, participate in regulating differentially expressed genes in the plant DNA damage response.

**Results:**

In this study, we used RNA-seq and ChIP-seq to show that differentially expressed genes (DEGs), in response to ionizing radiation (IR), might be involved in different pathways responsible for the DNA damage response. Moreover, chromatin structures associated with promoters, exons and intergenic regions are significantly affected by IR. Most importantly, either an individual mark or a certain chromatin state was found to be highly correlated with the expression of up-regulated genes. In contrast, only the chromatin states, as opposed to any individual marks tested, are related to the expression of the down-regulated genes.

**Conclusions:**

Our findings demonstrate that IR-related DEGs are modulated by distinct epigenetic mechanisms. Either chromatin states or distinct histone dynamics may act sequentially or in combination in regulating up-regulated genes, but the complex chromatin structure is mainly responsible for the expression of down-regulated genes. Thus, this study provides new insights into how up- and down-regulated genes are epigenetically regulated at the chromatin levels, thereby helping us to understand distinct epigenetic mechanisms that function in the plant DNA damage response.

**Electronic supplementary material:**

The online version of this article (10.1186/s12864-017-4172-x) contains supplementary material, which is available to authorized users.

## Background

Covalent histone modifications provide a platform for the recruitment of other non-histone proteins responsible for chromatin-related processes, thereby playing pivotal roles in diverse biological processes, including the regulation of gene expression and chromatin states [1]. DNA lesions and genomic instability occur ubiquitously and constantly due to the exposure of eukaryotic cells to intrinsic or environmental sources of DNA damage agents. To preserve genomic integrity, eukaryotic cells have evolved several elaborate safeguarding networks to perceive and counteract different types of damages [2]. Histone modifications have been reported as one of the key components of these networks, they serve as high-affinity binding sites or platforms to recruit trans-factors functioning in DNA repair and signaling propagation [3–6]. Emerging evidence demonstrates that local or global changes in the chromatin structure occur during the different stages of DNA damage and repair processes. Moreover, increasing evidence shows that several histone modifications and the corresponding writers or erasers have already been involved in the DNA damage response (DDR) directly or indirectly [7]. DNA damage-related alterations of histone marks in non-plant eukaryotes have been reported to include phosphorylation at various serine residues of H2A.X [8–11], acetylation at H3K9 and H3K56 [12] and methylation at H3K79 [13] and H4K20 [14].

The involvement of histone marks in DNA damage and repair processes has been extensively studied in mammalian and yeast systems. However, the role of histone marks in the plant DNA damage response is currently understudied. Ionizing radiation (IR) is a common DNA damaging agent used for producing plant mutagenesis [15]. Cytological or proteomic studies demonstrate the occurrence of dynamic changes in histone marks during IR-induced plant DNA damage [16, 17]. Currently, the global changes in transcription and histone modifications in response to IR treatment in plants are poorly understood. In this study, we primarily applied omics approaches (RNA-seq and ChIP-seq with a panel of commercial histone marks) to characterize global transcriptomic changes and dynamics of histone modifications in the rice genome post-IR. IR causes global changes in gene transcription, and differentially expressed genes (DEGs) might be involved in different pathways responsible for the DNA damage response. Among a panel of histone marks tested (seven marks, including H3K4me3, H3K36me3, H3K27me3, H3K4ac, H3K27ac, H3K4me1 and H4K12ac), the enrichment of H4K12ac and H3K4me3 is dramatically decreased post IR. Moreover, the IR-induced global chromatin changes mainly occur at the promoters, exons and intergenic regions. Most importantly, either an individual mark or a certain chromatin state with combined marks is found to be highly correlated with the expression of up-regulated genes, but only chromatin states, instead of any individual mark tested, are related to the expression of down-regulated genes. Thus, we profiled for the first time the global transcriptomic changes and dynamics of histone modifications under ^60^Co~γ ray radiation in rice. Our findings indicate distinct roles for chromatin states and distinct histone dynamics in regulating the gene expression in response to IR, thereby providing valuable references for epigenetic characterization of plants in response to other environmental stresses.

## Results

### Ionizing radiation causes a somatic DNA damage in rice

**Fig. 1:**
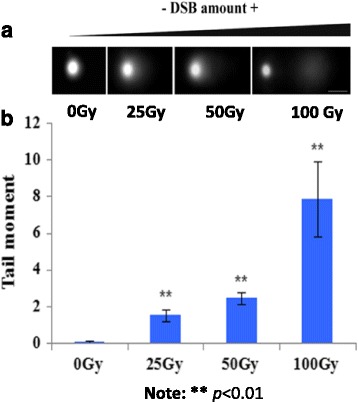
Comet assay of nuclei post-IR treatment with dosages as indicated. **a** Nuclei collected from the leaf tissue treated with 25, 50 and 100 Gy. Non-IR treated nuclei (0 Gy) were used as controls. **b** The comet tail moment values from the leaf tissue post-IR treated with dosages as indicated (0, 25, 50 and 100 Gy), respectively, were analyzed using comet assay software project (CASP). One hundred typical nuclei per treatment were selected for the statistical analysis. A statistical analysis was performed using analysis of variance, where **p < 0.01. The *x*-axis represents the IR dosage; the y-axis represents the tail moment value

To investigate the effect of ^60^Co~γ rays (ionizing radiation, IR) on somatic DNA damage in rice, we used 7-day-old sub-cultured rice seedlings (*Oryza sativa, subsp. japonica.l*) to perform the IR treatment with dosages of 0, 25, 50 and 100Gy. To test the degree of DNA damage induced by IR, we performed the comet assay under a neutral condition. We clearly observed the comet tail from nuclei extracted from the leaf tissue post-IR. By contrast, the comet tail was nearly invisible in the control nuclei (Fig. 1a). We then measured the comet tail moment value, which represents the level of DNA damage, using 100 typical IR-treated nuclei. As expected, the comet tail moment value exhibited a dose-dependent increment. The comet tail moment value from IR-treated nuclei was significantly higher than non-radiated control (*p*-value = 3.67E-61, student’s t-test) (Fig. 1b). For example, the comet tail moment value related to the 100-Gy treatment increased three times more than the one associated with the 25-Gy treatment. In agreement with the comet assay, the shoot length after 14-day culture post-IR displayed a dose-dependent decrease. Specifically, we found that 100-Gy was a lethal dose for the cultured seedlings since the seedlings with 100-Gy treatment displayed severe phenotypic defects, and some of the seedlings completely died after an additional 14-day culture post-IR (Additional file 1: Figure S1). Thus, these results indicate that the exposure to gamma radiation causes a dose-dependent DNA damage in rice.

**Fig. 2:**
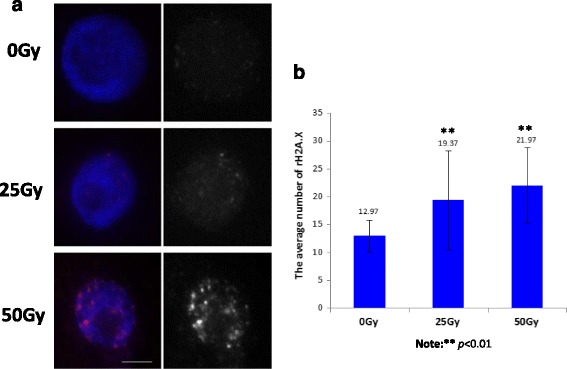
Immunofluorescence assay of γ-H2A.X phosphorylation foci after 50 Gy treatment. **a** Root tips were collected from the seedlings 12 h post-IR treatment with the dosages as indicated (0, 25, 50 Gy) for immunoassay with the homemade anti-γ-H2AX phosphorylation antibody. DAPI stained nuclei and immunosignals derived from anti-γ-H2AX phosphorylation antibody were observed and recorded under a fluorescence microscope (Olympus DP80). The merged colorful images were generated as indicated. The blue signal was derived from DAPI stained nuclei; the black and white image was generated from the nuclei stained with anti-γ-H2AX phosphorylation antibody with IR treatments as indicated. Bar, 10 μm. **b** The statistical assay of γH2A.X immunosignals. Foci of γH2A.X immunosignals were counted from 30 individual nuclei, which were prepared from root tips of the seedling post-IR treatment with indicated dosages (0, 25, 50 Gy) for 12 h. A statistical analysis was performed using the *t*-test, where **p < 0.01. The *x*-axis represents the IR dosage; the y-axis represents the average number of γH2A.X foci

The presence of phosphorylation of H2A.X at Ser 139 (γ-H2AX) is considered as a specific and sensitive molecular marker, which is highly associated with DDR in both mammalian and plant genomes [18–21]. To monitor the presence of γ-H2A.X phosphorylation post-IR in rice nuclei, we performed immunofluorescence assay on the nuclei isolated from root tips using the well characterized homemade antibody against γ-H2A.X phosphorylation. As expected, γ-H2A.X foci displayed a dose-dependent increase (Fig. 2a), which was further confirmed by the average number of γ-H2A.X foci counted from 30 nuclei (Fig. 2b). Consistent with results from the comet assay, the immunofluorescence result further indicates that DNA damage occurs after IR.

### Transcriptional changes are induced by ionizing radiation

To investigate the impact of IR on global changes in gene expression in the rice genome, we collected the leaf tissue from the plants with 30 min post-irradiation and extracted total RNA for RNA-seq. We found that the plants under 50 Gy treatment showed more differentially expressed genes than those treated with 25 Gy (3839 vs.2254). To further characterize the global transcriptional changes post-IR, we re-performed RNA-seq with two biological replicate using plants with 0 and 50 Gy treatments. The Pearson Correlation Coefficients (r) corresponding to biological replicates from 0 and 50 Gy were 0.958075 and 0.9658 (Additional file 2: Figure S2a), respectively. Moreover, the correlation between two replicated data sets from the same treatment was higher than those between different treatments (Additional file 2: Figure S2b). These analyses demonstrate that the sequencing data sets are reliable for further analysis. We identified 3839 differentially expressed genes with a fold change equal to or greater than 2, which included 2127 up-regulated and 1712 down-regulated genes (Additional file 3: Table S1). To test the accuracy of the DEGs identified by the sequencing data, we randomly selected 10 up-regulated and 8 down-regulated DEGs (18 total genes) for RT-PCR validation. We found that 17 out of 18 genes were consistent with the above mentioned computational identification (Additional file 4: Table S2), indicating a high accuracy of the computational DEGs identification.

**Fig. 3:**
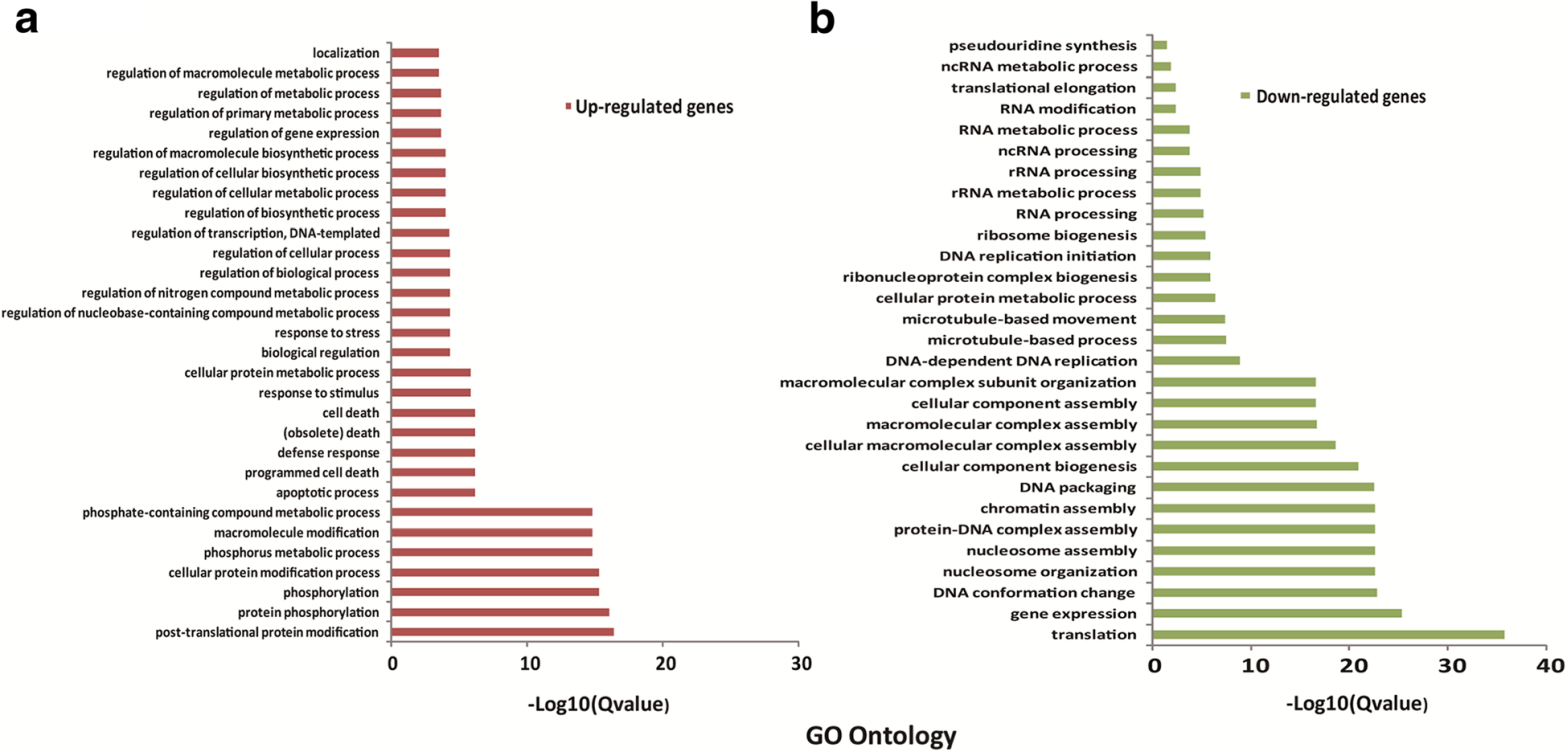
Gene ontology analysis of differentially expressed genes. RNA-seq data sets generated from the leaf tissue with 0 and 50 Gy treatments were analyzed for identification of differentially expressed genes (DEGs) before/after IR treatments, which include up- and down-regulated genes. **a** Functional enrichments of the up-regulated genes; **b** Functional enrichments of down-regulated genes

To investigate whether there are any functional preferences between up- or down-regulated genes, we performed gene ontology (GO) term enrichment analysis. As expected, we found functional divergences between up- and down-regulated genes (Fig. 3, Additional file 5: Table S3). Briefly, the up-regulated genes showed a strong overrepresentation of GO terms functioning in biological metabolisms, response to stress or stimulus, defense response, cell death and protein modification, those functions are probably related to DNA damage response (Fig. 3a), whereas the down-regulated genes were mainly responsible for DNA replication, gene expression, nucleosome organization, chromatin assembly, DNA packaging and cellular component assembly (Fig. 3b).

In addition, we detected eight DEGs responsible for the DNA damage and DNA repair (Additional file 5: Table S3), for example, LOC_Os11g40150, LOC_Os12g31370 and LOC_Os11g04954 are putative DNA repair protein Rad51, LOC_Os01g65890 is a putative DNA repair metallo-beta-lactamase, and LOC_Os07g32730 is a putative DNA repair protein rhp16, and LOC_Os02g47150 is the putative DNA topoisomerase 2.

Thus, IR causes global changes in gene transcriptions, and IR-induced DEGs might be involved in different pathways responsible for DNA damage response.

### Ionizing radiation causes dynamic changes of histone modifications

Histone modifications play vital roles in DNA damage responses, including the maintenance of genome integrity, by providing a landing platform for the recruitment of repair-related proteins [22]. To profile the effect of IR on chromatin-based changes, we performed ChIP experiments coupled with Illumina sequencing (ChIP-seq) using antibodies against seven histone marks, including H3K4me3, H3K36me3, H3K27me3, H3K4ac, H3K27ac, H3K4me1 and H4K12ac. We identified IR-induced differentially modified nucleosome regions (DMNR) associated with each mark before and after IR treatment using ChIPDiff software. We found that, among these seven marks tested, only H4K12ac- and H3K4me3-related nucleosomes exhibited visible changes between 0 and 50 Gy treatments. The number of DMNR with a fold change equal to or greater than 1.5 was 13,009 and 4453 for H4K12ac and H3K4me3 marks, respectively. We then performed a global peak calling for each mark, and found the total peak numbers were similar for H3K36me3, H3K27me3, H3K4ac and H3K27ac before and after IR treatment. However, the total peak counts for H4K12ac and H3K4me3 were dramatically decreased after IR, especially for H4K12ac (Table 1), which is consistent with the DMNR results mentioned above. The IR-induced dramatic decrease in the H4K12ac mark was further confirmed by Western blotting (Additional file 6: Figure S3), which indicates that the total H4K12ac loss occurs during IR treatment. Moreover, approximately 80–90% of peaks for H3K36me3, H3K27me3, H3K4ac and H3K27ac were shared between 0 and 50Gy treatments (Table 1), indicating the presence of IR-induced changes in histone marks. To further characterize IR-induced presence or absence of each mark, we calculated 0Gy- and 50 Gy-specific peaks for each mark, which corresponded to IR-induced loss (0 Gy-specific peaks) and gain (50 Gy-specific peaks) (Table 1). Indeed, when compared with the control (0Gy), we detected IR-induced presence/absence of each mark within the genome. For example, IR caused loss/gain of modified loci for H3K27me3 and H3K36me3 as 1211/808 and 470/1684, respectively. This analysis confirms that IR causes local or global changes in the chromatin structure, including an active (H4K12ac, H3K36me3, H3K4me3, H3K4ac and H3K27ac) or repressive (H3K27me3) chromatin state.

**Table 1:** Summary of ChIP-seq peaks from each mark between 0 and 50 Gy as indicated

Histone marks	Peak counts (0Gy)	Peak counts (50Gy)	Overlapping peaks	0Gy only peaks	50Gy only peaks
H3K27me3	11,990	10,309	9280	1211	808
H3K36me3	19,342	20,719	18,200	470	1684
H3K4me1	14,822	15,424	14,326	269	896
H3K4ac	20,525	20,630	18,163	2150	2249
H3K27ac	25,848	26,662	21,343	3221	4735
H3K4me3	32,782	27,530	26,483	6016	502
H4K12ac	20,466	9562	8669	11,762	644

**Fig. 4:**
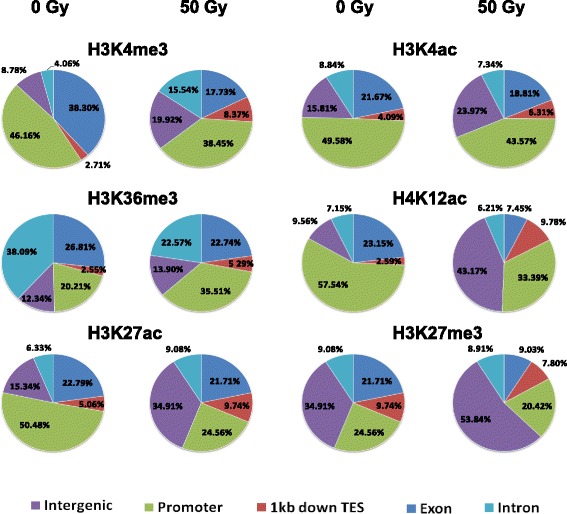
Genomic distribution of IR-induced ChIP-seq peaks from each indicated mark specific for 0 and 50 Gy. Each pie chart contains five sub-genomic regions, including promoters, exons, introns, 1 kb downstream of the TES and intergenic regions with at least 1 kb away from the TSS or TES. Each sub-genomic locus was indicated by using a different color

To investigate IR-induced changes in the genomic distribution of 0 Gy or 50 Gy-specific peaks for each mark tested in Table 1, we profiled 0 Gy or 50 Gy-specific peaks for each mark across five sub-genomic loci (promoters, exons, introns, 1 kb downstream of the TES and intergenic regions with at least 1 kb away from either the TSS or the TES of any genes) (Fig. 4). Our results demonstrated that a higher percentage of 0 Gy-specific peaks from H3K4me3, H3K4ac, H3K27ac, and H4K12ac marks, which disappeared after the 50 Gy treatment, were located at promoter and exon regions compared to other regions, while the percentage of H3K27me3 and H3K36me3 marks were higher at promoter and genic regions than other ones. By contrast, an apparent increase in the intergenic regions and a decrease in the promoter regions were observed in the distribution of H3K27me3, H3K4me3, H3K27ac, H3K4ac and H4K12ac marks post-IR when compared with the 0 Gy treatment (Fig. 4). The distribution of H3K27me3, H4K12ac and H3K4me3 in exons was decreased by 16%, 21% and 12%, respectively. Approximately 16% of H3K36me3 loss in introns occurred post-IR compared to those in the 0 Gy treatment. Collectively, all of the above analyses demonstrate that IR-induced global chromatin changes mainly occur at the promoters, exons and intergenic regions.

### Association between histone marks and differential gene transcription

**Fig. 5:**
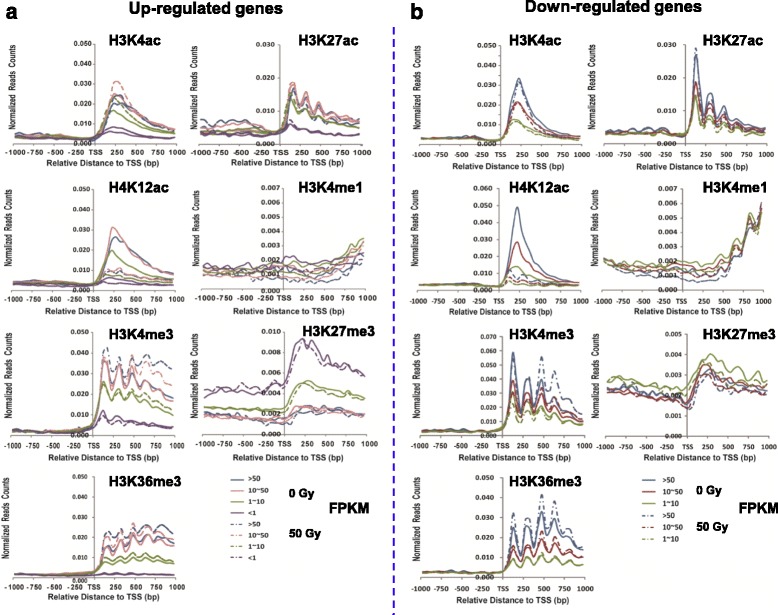
Distribution of histone marks across differentially expressed genes. **a** Profile of each indicated mark across the up-regulated genes (the dot and dash lines), divided into four sub-groups according to the FPKM value: less than 1, between 1 and 10, between 10 and 50, and greater than 50, and the corresponding untreated genes were used as controls (the solid lines); **b** Profile of each indicated mark across the down-regulated genes (the dot and dash lines), which were divided into three sub-groups according to the FPKM value: between 1 and 10, between 10 and 50, and greater than 50, and the corresponding untreated genes used as controls(the solid lines). The *x*-axis represents the position relative to TSS; the *y*-axis represents normalized reads counts from each mark indicated, indicating the enrichment of the corresponding mark

In general, active marks (H4K12ac, H3K27ac, H3K4ac, H3K36me3 and H3K4me3) are positively correlated with gene expression, whereas repressive marks (H3K27me3 and H3K9me2) are negatively correlated with gene expression in eukaryotes [23, 24]. To investigate if there exists any correlation between histone marks and the expression of IR-induced DEGs, we divided all up-regulated genes (2127) into four subgroups according to the FPKM values in the control (FPKM less than 1 (234 genes), between 1 and 10 (1081 genes), between 10 and 50 (577 genes), greater than 50(122 genes)). In general, when compared with untreated control, active marks (H3K4ac, H3K27ac, H3K4me3 and H3K36me3) were expectedly enriched more in the corresponding up-regulated genes (Fig. 5a), but the significant difference (Additional file 7: Table S4) in enrichment of the 1 kb downstream of the TSSs of the corresponding genes for each mark was only observed in the up-regulated genes with a certain expression levels (FPKM) (Additional file 8: Figure S4a). Consistent with the global decrease of H4K12ac post IR (Additional file 6: Figure S3), the enrichment of H4K12ac was dramatically decreased in the corresponding up- and down-regulated genes (Additional file 8: Figure S4). After a closer look, we observed a differential distribution of H3K4me3 within four subgroups of up-regulated genes. Compared to the untreated control, H3K4me3 was less enriched in genes with FPKM less than 1; there was no significant difference in genes with FPKM between 1 and 10, but H3K4me3 was more enriched in the other two subgroups with FPKM higher than 10 (Fig. 5a, Additional file 8: Figure S4a). This result indicates that H3K4me3 might be primarily responsible for regulating highly expressed up-regulated genes. Similarly, we divided all down-regulated genes into three categories according to the FPKM value (class I: between1 and 10, class II: between 10 and 50, and class III: greater than 50). After profiling each mark across down-regulated genes, we did not observe any consistent correlation between the enrichment of active/repressive marks and the down-regulated genes (Fig. 5b). Most marks were opposite to the general notion about marks tested and global gene expression. A significant difference of enrichment in the down-regulated genes with each expression level was observed in H3K4ac, H3K27ac, H4K12ac, H3K4me1 and H3K27me3, but H3K36me3 was significantly enriched the down-regulated genes with FPKM greater than 50, and H3K4me3 exhibited no difference in the down-regulated genes with FPKM between 10 and 50 (Additional file 8: Figure S4b; Additional file 9: Table S5). Therefore, a general positive correlation was observed between active marks and up-regulated genes, whereas no consistent trend existed between active/repressive marks and down-regulated genes. This analysis indicates that the presence of histone marks was mainly responsible for regulating IR-induced up-regulated genes. However, down-regulated genes might be regulated in a different way, which needs to be further investigated.

### Coordination of histone marks in regulating gene expression during ionizing radiation

**Fig. 6:**
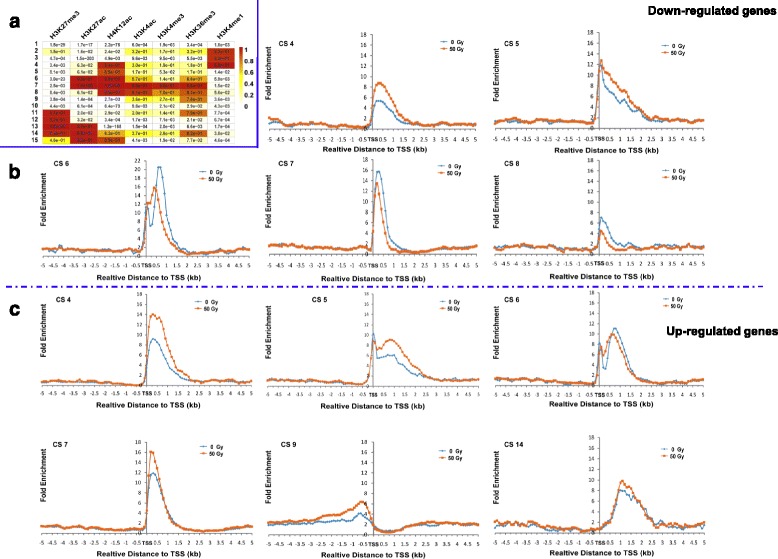
Distribution of chromatin states across 5 kb up- and down-stream of the TSS of differentially expressed genes. **a** Chromatin states analyzed by ChromHMM. The genome-wide chromatin state (CS) was divided into fifteen sub-groups according to the combination of 7 marks as indicated in each group. **b** Distribution of indicated CS across the control and the corresponding down-regulated genes. The *x*-axis represents the position relative to the TSS; the *y*-axis represents fold enrichment of the corresponding CS. **c** Distribution of indicated CS across the control and the corresponding up-regulated genes. The *x*-axis represents the position relative to the TSS; the *y*-axis represents fold enrichment of the corresponding CS. The blue solid line for separating (**a**) and (**b**), and the dot and dash line for separating (**b**) and (**c**)

The chromatin state, which comprises combinatorial marks rather than a single mark, has been reported to confer distinct roles in regulating gene transcription within various eukaryotic genomes [25–27]. The failure to detect any correlation between marks and down-regulated genes prompted us to suspect that the combination of marks, rather than an individual one, might facilitate the regulation of differentially expressed genes related to DNA damage responses. To test this possibility, we performed ChromHMM analysis and divided the genome-wide chromatin states (CSs) into fifteen subgroups according to the combination of 7 marks, as indicated in each group (Fig. 6a). Specifically, CS2 and CS3: overrepresentation of repressive mark H3K27me3; CS4: consisting of bivalent marks H3K4me3 and H3K27me3; CS6 and CS15: co-existence of H3K4me3 and H3K36me3; CS7: containing H3K4me3, H3K36me3, H3K4ac, H3K27ac and H4K12ac; CS8: the dominance of H3K4me3, H3K4ac and H3K27ac; CS11 and CS12: the dominance of H3K4me1; CS13:co-existence of H3K4me1 and H3K36me3; CS14: co-existence of H3K4me1, H3K36me3 and H3K27ac; in contrast, CS1 and CS10: lack of any marks tested.

To further investigate how each CS affect the DEGs, we specifically looked into the distribution of CSs across 5 kb upstream and downstream of the TSS related to up- and down-regulated genes (Additional file 10: Figure S5). Among all CSs tested, we observed that CS4, CS5, CS7, CS9 and CS14, which mainly consisted of active marks, displayed a positive correlation with the expression of up-regulated genes (Fig. 6c). Similarly, the enrichment of CS6, CS7 and CS8 was highly correlated with the expression of down-regulated genes (Fig. 6b). CS6 exhibited two distinct enriched regions, immediately flanking the TSS and the gene body, across the down- and up-regulated genes (Fig. 6b and c). This profile is consistent with the overrepresentation of H3K36me3 and H3K4me3 marks in the CS6, representing more enriched in gene body and the immediate downstream of the TSS (Fig. 5), respectively. In general, CS5 and CS6 displayed a distinct relationship between up- and down-regulated genes. CS5 enrichment did not display the much change in the down-regulated genes (Fig.6b), but it was more enriched in the up-regulated genes (Fig. 6c); By contrast, no visible change in CS6 enrichment was observed in the up-regulated genes, but it was indeed less enriched in the genic regions of the down-regulated genes. After a closer look, we found that an IR-induced elevated CS6 signal appeared at around the 150 bp immediately downstream of the TSS, which was possibly caused by the IR-induced enrichment of H3K4me3 in the region. This result indicates that CS5 and CS6 might play distinct functions in regulating these genes (Fig. 6). As expected, CS4, consisting of bivalent marks, H3K4me3 and H3K27me3, showed enrichments in both up- and down-regulated genes, indicating a dual role of CS4 in regulating DEGs by promoting expression of up-regulated gene, but repressing the expression of down-regulated genes (Fig. 6). Therefore, the relationship between an individual mark and DEGs indicates that IR-induced changes in chromatin states and distinct histone marks may act sequentially or in combination for regulating the up-regulated genes, but a certain chromatin state functioned in regulating down-regulated genes. Our findings suggest that a distinct chromatin-based mechanism might be responsible for the expression of up- and down-regulated genes during the DNA damage response.

## Discussion

### Differential expression of genes in response to ionizing radiation

Sessile plants have developed protective and/or repair mechanisms essential for detection and tolerance to wide ranges of environmental stimuli, ultimately ensuring their survival and propagation. Thousands of genes undergo transcriptional changes during the plants’ response to individual or combined biotic and abiotic stresses, such as drought, high salinity, low or high temperature, flooding, etc. [28–30]. Approximately 5800 rice genes were differentially expressed during drought stress [31, 32], and most of the differentially expressed genes in response to drought are temporally and spatially regulated [32]. Generally, stress-related variation in gene expression functions in various biological processes, thereby affecting physiological or morphological changes during the stress response. Thus, transcriptional changes are adapted mechanisms facilitating plant consecutive adaptation and genome evolution during environmental stress responses.

It is still unclear how IR affects global changes in gene transcription, thereby causing phenotypic defects in plants. Our transcriptional analysis demonstrates that approximately 3800 genes were differentially expressed with a fold-change equal to or greater than two in response to IR. It is logical to detect IR-induced global transcriptional changes because gamma-rays can produce high-energy leading to DNA fragmentation (double-strand break: DSB). Moreover, IR can induce secondary DNA ionization through the induction of oxidation of by-products. Thus, IR treatment produces both direct and indirect endogenous stress to plants [15]. Consistent with defects in phenotypic changes in seedlings due to IR treatment, biological processes, such as DNA replication, nucleosome assembly and DNA conformation change, are possibly down-regulated by IR. The effect of DNA-damaging agents on DNA replication and transcription, which ultimately results in mutagenesis and cell death, has been reported in mammalian cells [33, 34]. DNA replication processes inhibited by the accumulation of DNA lesions can activate the DNA repair mechanisms, thus letting damaged cells have enough time to repair damaged DNA and proceed DNA replication [35]. Inhibition of these processes causes defects in cell growth, which is consistent with phenotypic defects observed post-IR treatment. However, many up-regulated biological processes, such as biosynthetic, metabolic and cellular processes, may help to prevent against IR-induced cell damage. The involvement of phenylalanine in cellular damage has been found in soybean in response to ultraviolet light [36]. As a precursor for the synthesis of phyto-hormones, such as abscisic acid (ABA) and other chemicals, pyruvic acid plays important roles in mediating the function of chloroplast in plant stress responses, such as those in response to salt [37].Thus, IR-induced differential gene expression causes two-sided effects: negative and positive regulation of cell development and plant growth. In addition, mRNA-seq datasets generated in this study primarily represent the change of steady-state level of global transcripts during IR, global Run-on assay coupled to deep sequencing (GRO-seq) will more accurately assess IR-related changes in real-time transcription genome-wide, and thereby GRO-seq will provide more informative information about the impact of IR on global transcription than regular mRNA-seq.

### Dynamic changes of histone modifications in response to ionizing radiation

Dynamic or reversible alterations in an individual or combinatorial histone marks, such as methylation and acetylation, participate in regulating plant genes in response to abiotic stresses [38, 39], including drought [40–42], temperature [43, 44], and salinity [45]. DNA damage results in structural dynamics of localized chromatins around the damage sites, thereby facilitating the recruitment of proteins for DDR or the accessibility of the damaged DNA to the repair machinery [46]. The roles of histone marks in DDR have been intensively investigated in mammalian and yeast systems. The dynamics of histone marks serve as a platform for the recruitment of trans-factors functioning in DNA repair pathways and post-repair chromatin restoration [47–49]. It has been reported that the phosphorylation of H2A.X, methylation of H4K20 and acetylation of H4K16 are involved in damage signaling. Acetylation of H3K9 and H4K16 and the presence of the H2A(X) have been reported to function in regulating chromatin opening status. Moreover, H4 and H2B phosphorylation; H2A.X de-phosphorylation; acetylation at H3K14/23/56 and H4K5/8/12/16/91, de-acetylation of H3 and H4; and ubiquitination of H2AK119 have been linked with the chromatin restoration [50]. The impact of IR or other mutagens on the dynamics of histone modifications has not been well studied in plants so far. Through the investigation of a panel of histone marks post-IR, more or fewer dynamics in each mark tested are observed. The IR-induced mark-related dramatic changes frequently occur in several sub-genomic regions: except for H3K36me3 which is increased in promoters, the other marks are decreased in promoters and exons, but increased in intergenic regions. The variation in mark distribution is observed in introns, with a significant increase for H3K4me3, but a dramatic reduction for H3K36me3. Strikingly, the enrichment of H4K12ac and H3K4me3, which are usually linked with chromatin opening and promoting transcription of active genes under the normal condition, is decreased dramatically post IR. And both have a similar trend of IR related dynamic, a dramatic increase in intergenic regions, but a significant decrease in exons and promoters. These dynamics indicate that IR indeed causes structural changes of local chromatins, especially condensation of promoters and de-condensation of intergenic regions. These changes possibly facilitate the regulation of gene transcription for DDR in plants. Similarly, DNA damage-related dynamics of histone marks have been reported in eukaryotes before. Acetylation of diverse histone residues generally induces changes in the electrostatic interaction between modified histone and underlying DNA, and causes higher-order folding of the resulting chromatins, thereby histone acetylation is usually associated with relaxation of chromation structure and facilitates gene activation in eukaryotic genomes [51]. Acetylation level within the genome is regulated by specific writers (histone acetyltransferases) and erasers (histone deacetylases), which interacts with distinct coactivators or co-repressors within diverse chromatin complexes [52]. Thus, acetylation of specific histone residues might function in distinct transcriptional status of the underlying chromatins. A biphasic profile of H4K16 acetylation at DNA damage, decrease in early stage but increase in later stage, further indicates dynamics of localized chromatin structure in response to DNA damage [53]. A time point-dependent change of histone marks (H3K9me2 and H4K5ac), the signal intensity of each mark decreases at 48 h but increases at 72 h post treatment, has been reported in the cytological investigation of gamma-ray treated barley nuclei. Similar to our findings of the increase in H3K4ac and H3K27ac and decrease in H4K12ac, a global decrease in H4 acetylation and increase in H3 acetylation have been detected in *Arabidopsis* in response to X-ray related DNA damage [17]. Thus, the dynamics of histone marks in response to environmental stimuli universally occur among eukaryotic genomes. In human, H4K12ac may serve as an epigenetic mediator to involve in estrogen receptor-alpha activity via recruitment of BRD4 [54]. H4K12ac functions in developmental processes, such as spermiogenesis, through binding to promoters of developmentally important genes or overlapping with CTCF binding sites [55, 56]. However, how H4K12ac involves in DDR in plant genomes is still unclear so far, which needs further investigation.

### Involvement of histone marks in the differential regulation of up- and down-regulated genes

Growing evidence shows that the coordination of differential gene expression and chromatin dynamics facilitates the plants to respond or adapt to environmental stresses. The differential presentation of H3K4me3 in rice genes with different expression levels occurs under drought conditions [31]. However, there is a lack of detailed evidence showing how chromatin dynamics are involved in regulating DEGs. Moreover, it is still unclear how the actions of combined histone marks (chromatin states) regulate gene expression in plants’ responses to environmental stresses. Through profiling a panel of histone marks across DEGs during IR treatment, an individual mark or a certain chromatin state containing a set of marks is found to be highly correlated with the expression of up-regulated genes; however, only chromatin states, instead of most individual marks tested except that the dramatic decrease in H4K12ac might be responsible for down-regulation of genes post-IR, is related to the expression of down-regulated genes. This analysis indicates that up- and down-regulated genes are differentially regulated. In addition, the chromatin containing the bivalent marks (CS4) H3K4me3 and H3K37me3 is highly enriched in both up- and down-regulated genes, indicating H3K4me3 and H3K37me3, with functional exclusion of each other, function independently in promoting the expression of up-regulated genes and repressing the expression of down-regulated genes. Thus, the combined chromatin state displays dual roles in regulating gene expression in response to IR. Similar findings have been previously reported in other environmental stimuli. A combination of H3K27me3 with its antagonistic marks H3K4me3 or H3K36me3 confers distinct roles in regulation of genes response to different stresses. For example, H3K27me3 and H3K4me3 function independently in modulating the expression of several individual memory genes in the dehydration response [57]. A functional antagonism between H3K36me3 and H3K37me3 acts as an epigenetic switch for regulating the *Arabidopsis FLC* gene during vernalization [58]. Thus, changes in chromatin states and distinct histone dynamics may act sequentially or in combination for the up-regulation of genes. However, a complex chromatin structure might be necessary to regulate the down-regulated genes.

## Conclusions

This is the first study for reporting global transcriptional changes after IR treatment. In addition, through integrating RNA-seq and ChIP-seq, we found that chromatin dynamics occur during IR. Most importantly, we found that either chromatin states or distinct histone dynamics may act sequentially or in combination in regulating up-regulated genes, but the complex chromatin structure is mainly responsible for the expression of down-regulated genes. Thus, this study provides new insights into how up- and down-regulated genes are epigenetically regulated at chromatin levels, thereby helping us to understand distinct epigenetic mechanisms that function in the plant DNA damage response.

## Materials and methods

### Tissue culture and ^60^Co~γ ray radiation

Rice seedlings (*Oryza sativa*,Nipponbare) were cultured in glass test tubes using half-strength of MS medium supplemented with NAA under 26 °C with 13 h light/11 h dark cycle. Seven-day-cultured seedlings were collected to perform ^60^Co~γ ray radiation treatment (Nanjing Xiyue Irradiation Technology company in Nanjing Gaochun city, China) with dosages equivalent to 25, 50 and 100 Gy, respectively. Untreated controls (0 Gy) were collected at the time point as indicated in the context. All samples were collected and quick-frozen in liquid nitrogen. Quick-frozen tissues were stored at −80 °C until use.

### Comet assay

The γ-ray-radiated leaf tissue and non-treated control were used for the preparation of nuclei. A single cell gel electrophoresis assay (Comet) for DSB detection was performed as previously described with minor modifications [59]. Briefly, approximately 0.1 g leaf tissue was chopped in 100 μl of pre-chilled 1xPBS containing 50 mM EDTA, respectively. After incubation on ice for 5 min, the nuclei were filtered with 200 μm nylon mesh. Approximately 30 μl of filtered nuclei completely mixed with 70 μl of 1% low-melting-point agarose were spread on the microscope slide, which was pre-coated with 1% regular agarose. After solidification at 4 °C for 10 min, the nuclei were subjected to lysis by incubation with a high salt solution (2.5 M NaCl, 10 mM Tris–HCl pH 7.5, 100 mM EDTA) at 4 °C for 1 h and followed by neutralization at 4 °C using 1xTBE three times for 5 min each. The neutralized nuclei were electrophoresised in pre-chilled 1xTBE for 8 min at RT. After electrophoresis, the slides were kept in 1% of Triton X-100 for 10 min to clear the starch grains from the gels and were then dehydrated in 70% and 90% ethanol for 5 min each, respectively. After air drying, the nuclei were stained with propidium iodide (PI, Sigma, Cat.# p4170), and the comet images were captured using a fluorescence microscope (Olympus DP80) equipped with a CCD camera. DNA damage signals were analyzed using the comet assay software project (CASP) software. The “tail moment” value, which is defined as the product of tail length and the percent of tailed DNA relative to the total DNA, was used to indicate the extent of DNA damage. One hundred typical nuclei were selected for statistical measurements at the time point indicated by the context. Each measurement was repeated at least three times.

### Quantitative RT-PCR (qRT-PCR) assay

Total RNA was extracted from γ-ray-radiated and control leaf tissues using RNeasy plant Mini kit (Qiagen, Cat#74904). The extracted total RNA was treated with DNaseI at 37 °C for 30 min to completely remove genomic DNA contamination. After DNaseI treatment, the total RNA was reversely transcribed in the first-strand cDNA following the manual from SuperScript III Reverse Transcriptase kit (Cat.# 18,080–044, Thermo Fisher Scientific). qRT-PCR was performed using the Roche LightCycler480 Real-time system (Roche). The PCR program was conducted as follow: 95 °Cx30 sec, 40 cycles of 95 °Cx5 sec, 55–65°Cx5 sec and plateread, 65°Cx31 sec, 60 cycles of 65°Cx5 sec with 0.5 °C/cycle with ramp 0.5 °C/s and plateread. The qRT-PCR primer sequences were listed in the Additional file 4: Table S2.

### Immunofluorescence assay

Slides preparation and immuno-detection procedures were exactly followed as described by Zhang et al. [60]. Briefly, root tips collected from 12 h post-IR treatment with dosage as indicated were fixed with 4% of paraformaldehyde in PBS (pH 7.0) for 30 min at 4 °C. Fixed root tips were washed with pre-chilled 1xPBS three times, the root tips were then cut with single-sided razor blade, and put on the slide and covered with a coverslip for slide preparation using the squash method. The prepared slides were stored in −80 °C until use. The homemade rabbit anti-gamma H2A.X (phospho S139) antibody specific for rice was used in the immunoassay, which has already been well characterized before [61, 62] . After removing the cover glasses, the slides were incubated with 50–100 μl of 1xTNB (100 mM Tris-HCl, pH 7.5, 150 mM NaCl, and 0.5% blocking reagent) containing 1:10 diluted anti-gamma H2A.X at 37 °C for 2 h in a wet chamber. After washing in 1XPBS three times with 10 min each, the slides were incubated with goat anti-rabbit secondary antibody conjugated with Rhodamine at 37 °C for 30 min in the wet chamber. After washing in 1XPBS three times with 10 min each, the nuclei were counterstained with Vectorshield mounting medium containing DAPI (Vector laboratory, Cat.#:H-1200).The immuno-signals were captured digitally using fluorescence microscope (Olympus DP80).

### Data analysis

#### RNA-seq

Total RNA was extracted from γ-ray-radiated and control leaf tissues with time points as indicated in the context using RNeasy plant Mini kit (Qiagen, Cat#: 74,904). Two biological replicates from each treatment were used for the downstream preparation of RNA-seq libraries. All RNA-seq libraries were prepared and sequenced with 150 bp PE on Illumina Hiseq4000 platform. The clean sequencing data sets without any contamination of adapter sequences were analyzed using the previously described pipeline [63]. Briefly, TopHat software [64] was used for mapping sequencing reads to the rice reference genome of the version 7 pseudo-molecules (http://rice.plantbiology.msu.edu/pub/data/Eukaryotic_Projects/o_sativa/annotation_dbs/pseudomolecules/version_7.0/). Cufflinks [65] was used to detect the expression values (FPKM) of annotated genes in rice with parameter -G. Differentially expressed genes (DEGs) were calculated by Cuffdiff. All DEGs, including up- and down-regulated genes, were used for further analysis. The cutoff of DEGs were defined by using the standard as |log_2_^(fold change)^| ≥ 1 and *q*-value <0.05 (Additional file 5: Table S3).

GO enrichment analysis was calculated by using the software AgriGO (http://bioinfo.cau.edu.cn/agriGO/), which specifically focuses on agricultural species [66].To display the data conveniently, the redundant GO terms were removed by the REVIGO tool (http://revigo.irb.hr/) [67], with the default parameters for the *O. sativa* GO term background.

#### ChIP-seq and ChIP-qPCR assay

We generated the following seven ChIP-seq data sets using a previously described method [63]. Leaf tissue from 50 Gy-treated and control, which are the same as those used for RNA-seq, were used for the ChIP experiment. The key procedures for the ChIP experiment are as below: extraction of nuclei followed by MNase-based fragmentation, incubation of antibodies of interest with fragmented chromatins overnight at 4 °C, recovery and purification of antibody-bound DNA fragments for the downstream assay, such as ChIP-qPCR and sequencing library preparation. The ChIP-grade antibodies used as below, H3K4ac (Millipore, 07–539), H3K27ac (Abcam, ab4729), H3K27me3 (Millipore, 07–449), H3K36me3 (Abcam, ab9050), H4K12ac (Millipore, 07–595), H3K4me1 (Abcam, ab8895) and H3K4me3 (Abcam, ab8580). The ChIP-seq libraries were sequenced on Illumina Hiseq 4000 platform. All of the ChIP-seq data sets were analyzed using a previously described pipeline [63]. Briefly, the raw ChIP-seq was cleaned by completely removing the contamination of adapter sequences, the clean data was then mapped to the rice reference genome by the short reads mapping software bowtie1 [68] with the parameters as -a -m 1 --best --strata --chunkmbs 200 -X 800. Unique mappable reads were used to call significantly enriched peaks from each mark by Macs14 (version 1.4.2) [69], with parameter -t -c --wig --single -g 3.8e + 8 (Additional file 11: Table S6). Normalized reads counts of each mark tested were calculated as below, the upstream and downstream 1 kb of the TSSs of each gene in the specified gene groups was split into small windows containing 50 bp each, the total number of reads within each window was counted, then divided by the window length (bp) and the number of unique reads (Million). To profile the specified gene groups, the average value of the same order located relative to TSSs window were calculated. The genomic location of each reads was decided by the center point of the corresponding pair-end fragment.

To confirm the H4K12ac ChIP-seq data, we conducted a ChIP-qPCR assay following the ChIP experiment with anti-H4K12ac. Twelve up-regulated and ten down-regulated genes were randomly selected to design primers for ChIP-qPCR analysis (Additional file 12: Table S7). 18S gene was used as the internal control to normalize the starting amount of input and ChIPed DNA from 0 Gy and 50 Gy. One primer set was triplicated in the qPCR assay. The summary information of ChIP-qPCR assay was listed in the Additional file 12: Table S7.

#### Identification of differentially modified nucleosome regions (DMNRs)

The DMNRs were identified using ChIPDiff [70]by comparing the fold difference between the IR-treated data and the corresponding control. The configure file ‘config.txt’ was edited in our analysis, including the bin size as 1 kb, the threshold for confidence as 0.95 and the minimal fold change as 1.5 etc.

#### Chromatin states

ChromHMM [71], which is based on a multivariate Hidden Markov Model that explicitly models the presence or absence of each mark, was used to characterize the chromatin states in this study. 15 chromatin states through the LearnModel parameters were generated similarly as the previous publication [71], each state contains a distinct combination of seven marks tested, including H3K4ac, H3K27ac, H4K12ac, H3K27me3, H3K4me1, H3K4me3 and H3K36me3. The segmentation size of each CS was indicated in Additional file 13: Figure S6). The enrichment for each state around ±5 kb of the TSSs of DEGs was calculated by the program ‘Neighborhood Enrichment’, with parameter -b 100 -r 50 -l 50.

### Western blot assay

Total proteins were extracted from leaf tissue using protein extraction buffer (1 M Tris-HCl pH 7.5, 5 M NaCl, 0.1%NP40, 24% [*w*/*v*] Urea, 1 mM PMSF and 1 mM DTT). Approximately 40 μg of protein per sample was mixed with 2xloading buffer, and heated for 10 min at 95 °C. The total denatured proteins were fractionated using 12% SDS-PAGE gel followed by blot preparation using semi-dry for 1 h at 100 V. The blot was pre-blocked with 1xPBS supplied with 3% albumin for 2 h with constant shaking at RT. After pre-blocking, the blot was immediately transferred in 1xPBS with rabbit antibodies against H4K12ac (07–595, Millipore) in 1:4000–6000 dilution, respectively, and incubated at 4 °C overnight in the shaker. After sequentially washing three times with 1xPBS, and three times with 1xPBS plus 0.1% Tween for 10 min each, the blot was incubated in 1xPBS + 0.1%Tween with 1:10,000 diluted goat ant-rabbit secondary antibody conjugated with HRP for 2 h at RT. After washing three times in 1xPBS plus 0.1% Tween, the blot was developed, and the immunosignal was digitally recorded. For a relative comparison, the same blot was sequentially probed with rabbit antibody against H3 (Abcam, ab1791). Immuno-signals were quantified and analyzed using Clinx Chemi Analysis software.

## Additional files

Additional file 1: Figure S1. The phenotypic observation of seedlings post IR treatment. (a) Seven-day-sub-cultured seedlings were irradiated with 25, 50 and 100 Gy of ^60^Co-γ rays at 3.3 Gy/min, respectively, and allowed to grow for another 14 days for monitoring phenotypic changes, which were captured on the 14th day after irradiation. Non-IR treated nuclei (0 Gy) were used as controls. (b) Shoot lengths were measured and averaged from 30 seedlings irradiated with respective doses of 25, 50 and 100 Gy. Non-IR treated nuclei (0 Gy) were used as controls. A significance test was performed using analysis of variance, where **p < 0.01. (PDF 87 kb)

Additional file 2: Figure S2. An association analysis of biologically replicated RNA-seq data sets between 0 and 50 Gy. (a) An associated analysis of RNA-seq data sets from biological replicates was performed between 0 Gy (top panel) and 50 Gy (bottom panel). (b) A pair-wise heatmap was generated using four data sets as indicated to show the reliability of data sets from each treatment. (PDF 140 kb)

Additional file 3: Table S1. Summary of DEGs post-IR treatment. (XLS 1086 kb)

Additional file 4: Table S2. Summary of genes information for qRT-PCR. (PDF 40 kb)

Additional file 5: Table S3. Summary of GO terms corresponding to up- and down-regulated genes. (XLS 326 kb)

Additional file 6: Figure S3. Western blot assay. Total proteins extracted from 50 Gy treated and untreated leaf tissues, were fractionated and transferred onto the blot. The blot was incubated with primary rabbit antibodies against H4K12ac followed by detection with goat ant-rabbit secondary antibody conjugated with HRP. The blot was developed and the immunosignal was digitally recorded (the top panel). For relative comparison, the same blot was sequentially probed with rabbit antibody against H3 (Abcam, ab1791) (the bottom panel). (PDF 32 kb)

Additional file 7: Table S4. The *p*-value of Wilxcoxon rank-sum test (one side) for each mark distributed across up-regulated genes between 0 Gy and 50 Gy. (PDF 54 kb)

Additional file 8: Figure S4. Significance test of difference in the enrichment of each mark across DEGs with different expression levels (FPKM) between 0 Gy and 50 Gy. The box-plot showing the enrichment of each mark tested across DEGs with different expression levels (FPKM) between 0 Gy (the white box) and 50 Gy (the grey box). The Wilcoxon test (one side) was conducted to test the significance of difference in the enrichment of each marks across DEGs with different expression levels between 0 Gy and 50 Gy, where * *p*-value <0.05, ** *p*-value <0.01 in Wilcoxon test (one side) (a) The up-regulated genes between 0 Gy and 50 Gy were divided into four subgroups according to FPKM: less than 1, between 1 and 10, between 10 and 50, and greater than 50. (b) The down-regulated genes between 0 Gy and 50 Gy were divided into three subgroups according to FPKM: between 1 and 10, between 10 and 50, and greater than 50. The *x*-axis represents the FPKM value; the *y*-axis represents normalized read counts from each mark indicated, indicating the enrichment of the corresponding mark between 0Gy (the white box) and 50 Gy (the grey box). (PDF 478 kb)

Additional file 9: Table S5. The *p*-value of Wilxcoxon rank-sum test (one side) for each mark distributed across down-regulated genes between 0 Gy and 50 Gy. (PDF 54 kb)

Additional file 10: Figure S5. Distribution of chromatin statuses (CSs) across differentially expressed genes. The genome-wide chromatin state (CS) was divided into fifteen subgroups according to the combination of 7 marks as indicated in each group. (a) Distribution of chromatin states across control genes and the corresponding up-regulated genes with 5 kb up and down stream of the TSS. (b) Distribution of chromatin state across control genes and the corresponding down-regulated genes with 5 kb up and down-stream of the TSS. The *x*-axis represents the position relative to TSS; The *y*-axis represents fold enrichment, indicating the enrichment of the corresponding CS. (PDF 388 kb)

Additional file 11: Table S6. Summary of the sequencing mapping of RNA-seq and ChIP-seq data sets. (PDF 37 kb)

Additional file 12: Table S7. Summary of anti-H4K12ac based ChIP-qPCR assay. (PDF 83 kb)

Additional file 13: Figure S6. Segmentation size of each chromatin states (CS). The box-plot showing the length of chromatin state (CS) between 0 and 50 Gy.The *x*-axis represents a specific CS; The *y*-axis represents the length of chromatin states (bp). (PDF 247 kb)

## Abbreviations

ABA: abscisic acid
CASP: comet assay software project
CS: Chromatin state
DDR: DNA damage response
DEGs: differentially expressed genes
DMNR: modified nucleosome regions
DSB: double-strand break
GO: gene ontology
GRO-seq: global Run-on assay coupled to deep sequencing
IR: ionizing radiation
PI: propidium iodide
qRT-PCR: Quantitative RT-PCR
