# Supplementary figures and images for: Chromatin states responsible for the regulation of differentially expressed genes under 60Co~γ ray radiation in rice
Source: BMC Genomics. 2017 Oct 12;18:778. doi: 10.1186/s12864-017-4172-x (PMC5639768; doi:10.1186/s12864-017-4172-x)

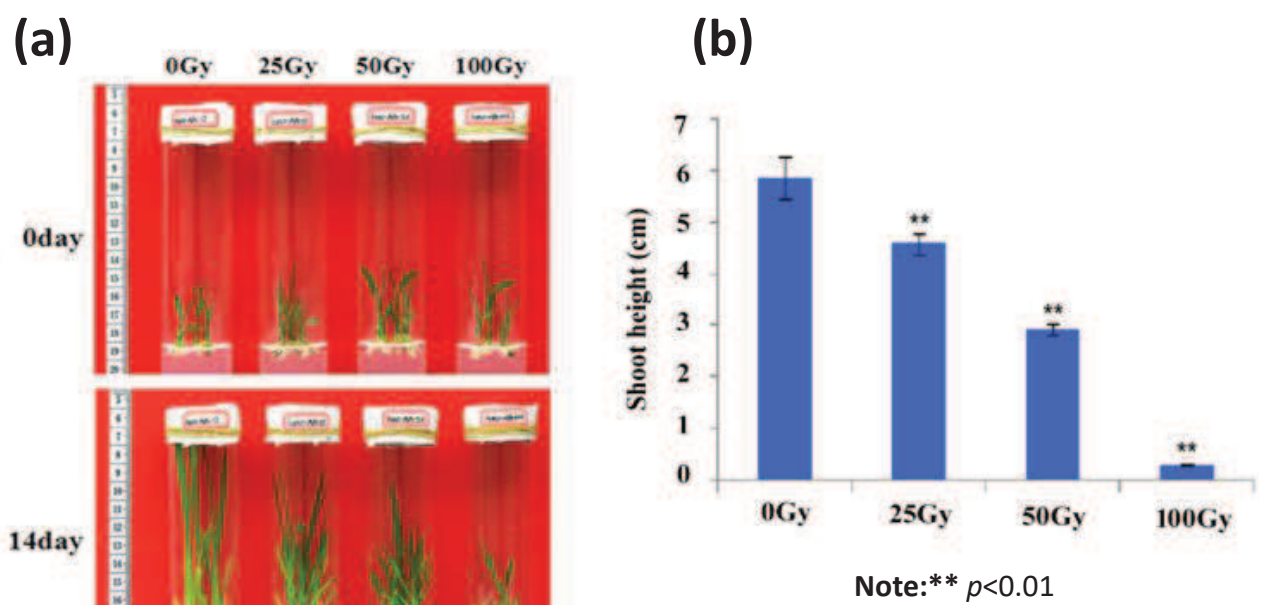

Fig. S1

Supplement: Supplementary file 1 — The phenotypic observation of seedlings post IR treatment. (a) Seven-day-sub-cultured seedlings were irradiated with 25, 50 and 100 Gy of 60Co-γ rays at 3.3 Gy/min, respectively, and allowed to grow for another 14 days for monitoring phenotypic changes, which were captured on the 14th day after irradiation. Non-IR treated nuclei (0 Gy) were used as controls. (b) Shoot lengths were measured and averaged from 30 seedlings irradiated with respective doses of 25, 50 and 100 Gy. Non-IR treated nuclei (0 Gy) were used as controls. A significance test was performed using analysis of variance, where **p < 0.01. (PDF 87 kb) [file 12864_2017_4172_MOESM1_ESM.pdf]

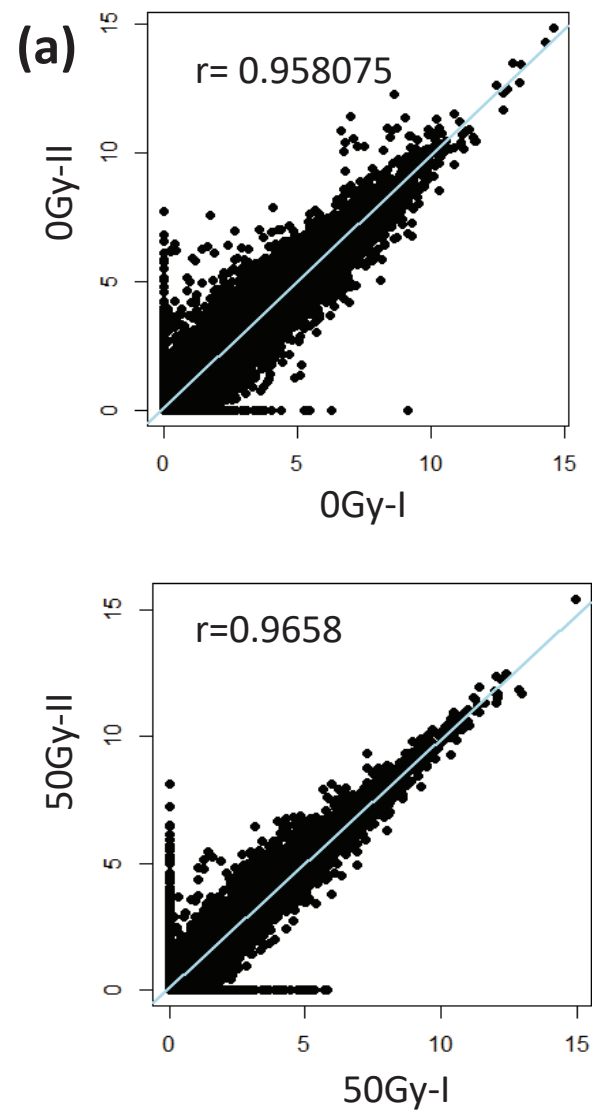

**(b)**

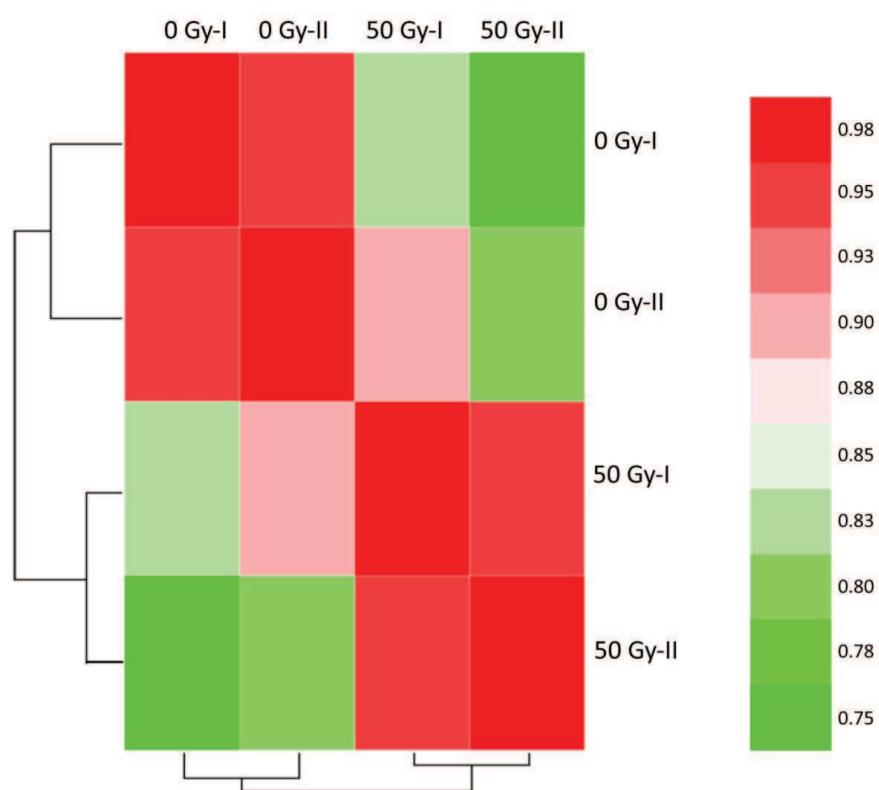

**Fig. S2**

Supplement: Supplementary file 2 — An association analysis of biologically replicated RNA-seq data sets between 0 and 50 Gy. (a) An associated analysis of RNA-seq data sets from biological replicates was performed between 0 Gy (top panel) and 50 Gy (bottom panel). (b) A pair-wise heatmap was generated using four data sets as indicated to show the reliability of data sets from each treatment. (PDF 140 kb) [file 12864_2017_4172_MOESM2_ESM.pdf]

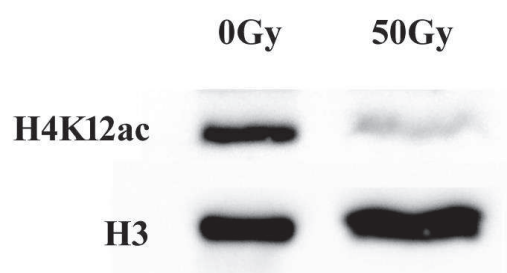

**Fig. S3**

Supplement: Supplementary file 6 — Western blot assay. Total proteins extracted from 50 Gy treated and untreated leaf tissues, were fractionated and transferred onto the blot. The blot was incubated with primary rabbit antibodies against H4K12ac followed by detection with goat ant-rabbit secondary antibody conjugated with HRP. The blot was developed and the immunosignal was digitally recorded (the top panel). For relative comparison, the same blot was sequentially probed with rabbit antibody against H3 (Abcam, ab1791) (the bottom panel). (PDF 32 kb) [file 12864_2017_4172_MOESM6_ESM.pdf]

**(a)** Up-regulated genes

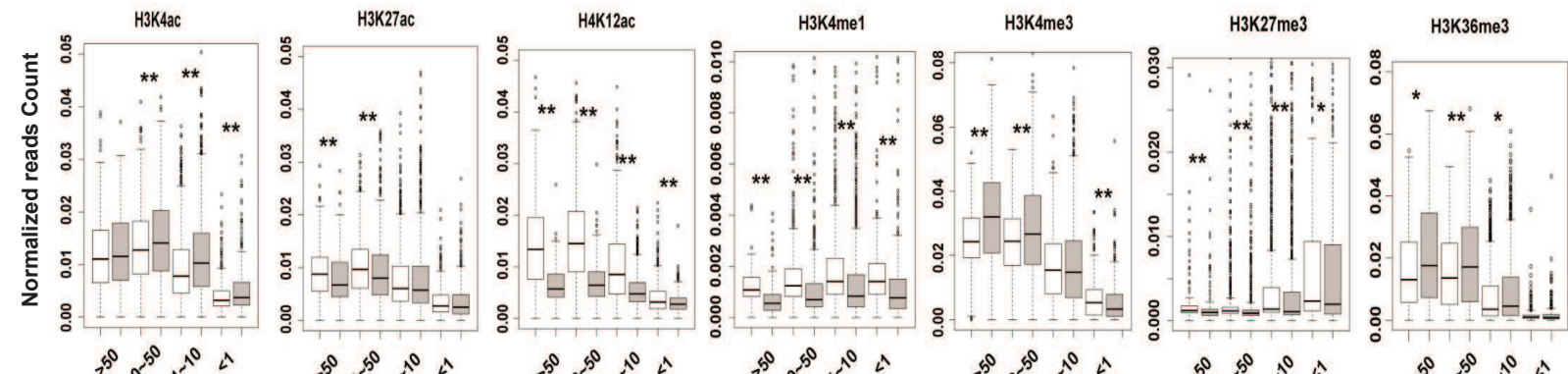

**(b)** Down-regulated genes

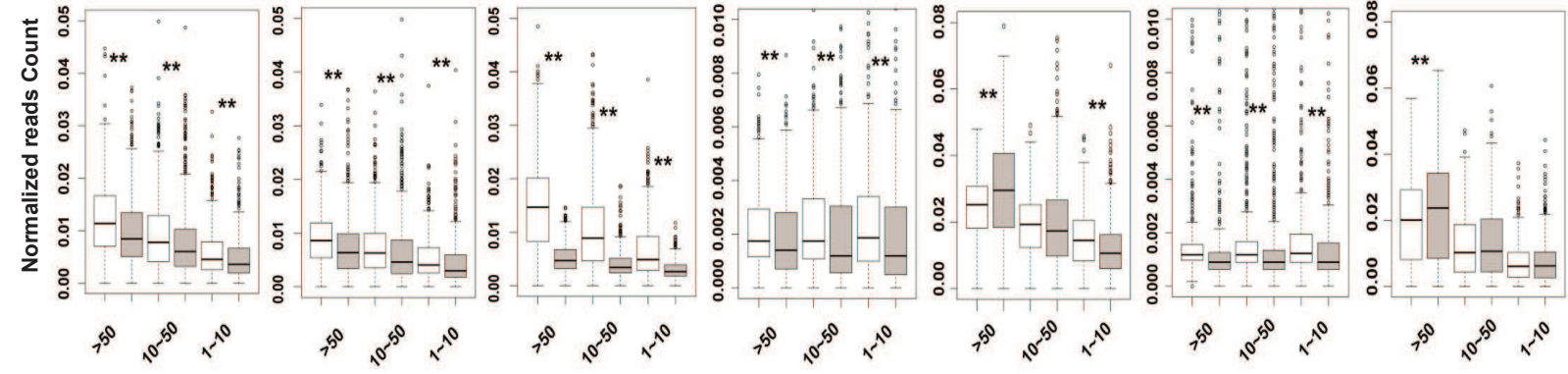

\*  $p$ -value < 0.05, \*\*  $p$ -value < 0.01

**Fig. S4**

Supplement: Supplementary file 8 — Significance test of difference in the enrichment of each mark across DEGs with different expression levels (FPKM) between 0 Gy and 50 Gy. The box-plot showing the enrichment of each mark tested across DEGs with different expression levels (FPKM) between 0 Gy (the white box) and 50 Gy (the grey box). The Wilcoxon test (one side) was conducted to test the significance of difference in the enrichment of each marks across DEGs with different expression levels between 0 Gy and 50 Gy, where * p-value <0.05, ** p-value <0.01 in Wilcoxon test (one side) (a) The up-regulated genes between 0 Gy and 50 Gy were divided into four subgroups according to FPKM: less than 1, between 1 and 10, between 10 and 50, and greater than 50. (b) The down-regulated genes between 0 Gy and 50 Gy were divided into three subgroups according to FPKM: between 1 and 10, between 10 and 50, and greater than 50. The x-axis represents the FPKM value; the y-axis represents normalized read counts from each mark indicated, indicating the enrichment of the corresponding mark between 0Gy (the white box) and 50 Gy (the grey box). (PDF 478 kb) [file 12864_2017_4172_MOESM8_ESM.pdf]

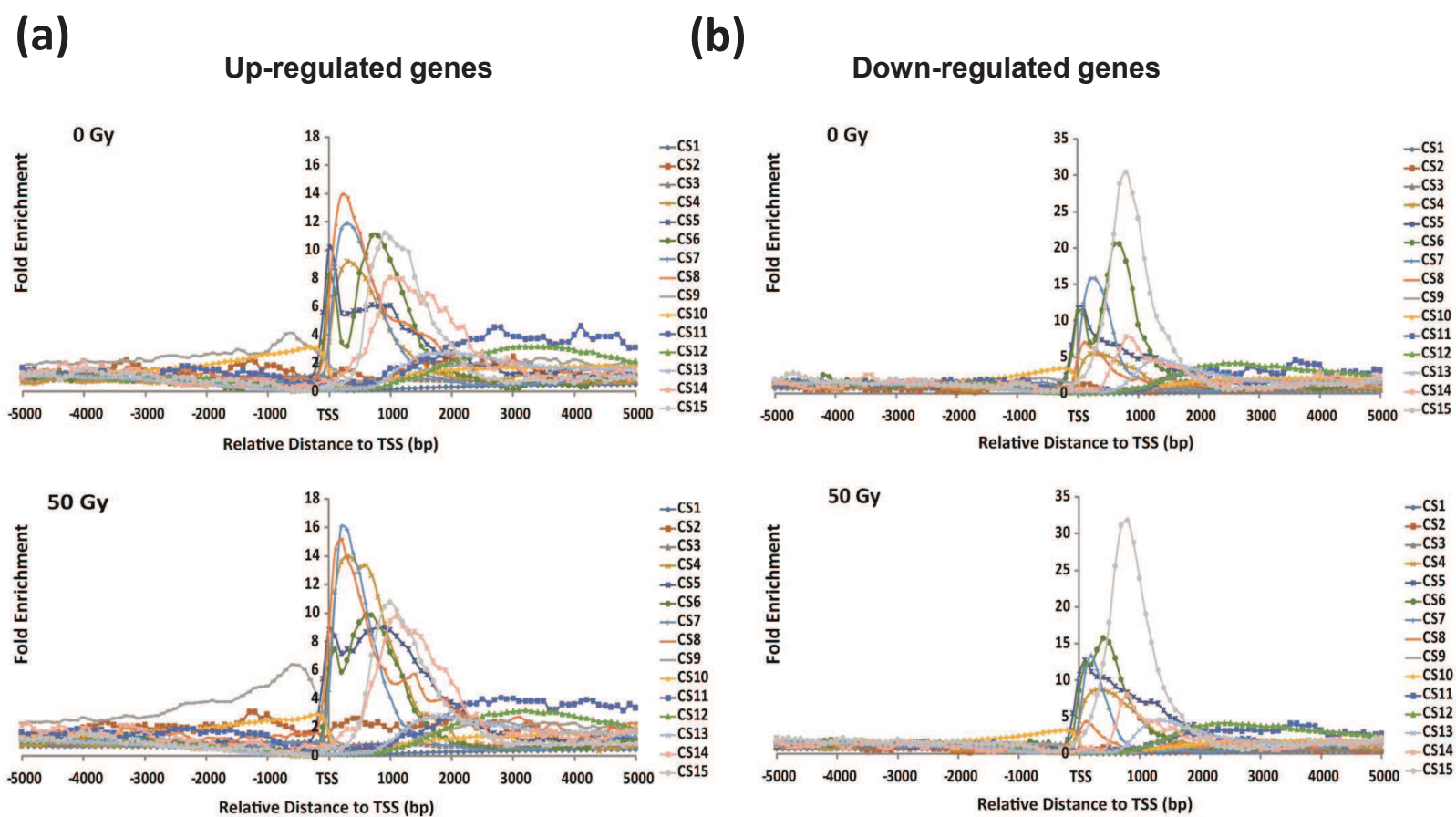

**Fig. S5**

Supplement: Supplementary file 10 — Distribution of chromatin statuses (CSs) across differentially expressed genes. The genome-wide chromatin state (CS) was divided into fifteen subgroups according to the combination of 7 marks as indicated in each group. (a) Distribution of chromatin states across control genes and the corresponding up-regulated genes with 5 kb up and down stream of the TSS. (b) Distribution of chromatin state across control genes and the corresponding down-regulated genes with 5 kb up and down-stream of the TSS. The x-axis represents the position relative to TSS; The y-axis represents fold enrichment, indicating the enrichment of the corresponding CS. (PDF 388 kb) [file 12864_2017_4172_MOESM10_ESM.pdf]

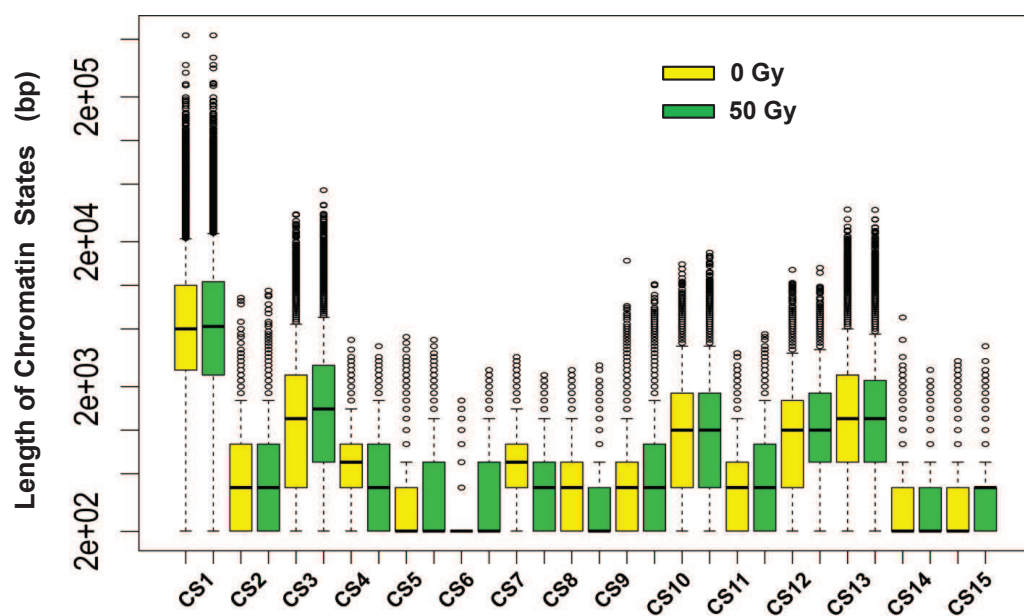

Fig. S6

Supplement: Supplementary file 13 — Segmentation size of each chromatin states (CS). The box-plot showing the length of chromatin state (CS) between 0 and 50 Gy.The x-axis represents a specific CS; The y-axis represents the length of chromatin states (bp). (PDF 247 kb) [file 12864_2017_4172_MOESM13_ESM.pdf]
