# Supplementary material for: Chromatin states responsible for the regulation of differentially expressed genes under 60Co~γ ray radiation in rice
Source: BMC Genomics. 2017 Oct 12;18:778. doi: 10.1186/s12864-017-4172-x (PMC5639768; doi:10.1186/s12864-017-4172-x)
Supplement: Supplementary file 4 — Summary of genes information for qRT-PCR. (PDF 40 kb) [file 12864_2017_4172_MOESM4_ESM.pdf]

Table S2: Summary of genes information for qRT-PCR

Locus number	Forward primer	Reverse primer	qRT-PCR	RNA-seq
LOC_Os04g33050	ATAGCATGCCACAACAAGGAGA	TAAGAGCTTTGGATGGTGGCAT	+	+
LOC_Os01g65890	TCTTGGTCACGAAGAGATCCTA	GTGATGATTTCTGGGGCTATGA	+	+
LOC_Os02g57350	ATGGGGAAGAAGAGCAAGAACG	GATCTTGTCCTTGCAGCCATTG	+	+
LOC_Os05g15770	TGTCGCACAAGGATCTCTACTA	GTCTTCTTGTCCCAGTACCTG	+	+
LOC_Os10g11500	ACTACCACTACGACACCAACAC	TCGTAGTTGCAGGTGATGAACA	+	+
LOC_Os05g34150	GAGGACCTCGGCAACAAGAG	GTACTGGAGGATGATGACGGAC	+	+
LOC_Os10g18370	GAGGAGTGCTTCTTCCTGCTG	GAAGAAGCGGAAGTCCCTCAC	+	+
LOC_Os08g33100	GACAACAAGAAGACGCGGATC	GAGGAGCAGGTTGTGGATGTT	+	+
LOC_Os08g06110.2	GCTCTTCATGTGGTTCCAACAC	CCATGAATCACTTGTGCTTGCA	+	+
LOC_Os12g31370	TGGTGGACGCTTGGATTGAT	CAGGATTCCAGGGCGCTAT	+	+
LOC_Os07g30670.1	TGCATCGTCGAGGTAGTTGAAG	CACGCCAATCTCCATGTCTTTG	−	−
LOC_Os01g50622.1	TGCGGATTACAGAAATGGTCCA	TAGGAACCTTACACCGGAAAGC	−	−
LOC_Os03g38950.1	CTTCCCAGACGAAAACGTTGTC	GAATCGGAATCCATTGGGCATG	−	−
LOC_Os01g01340.1	ACAGCTCTAACATCTCGGTGTT	TCGTCGTAGGATAGGTAGTCCC	−	−
LOC_Os08g33100	GACAACAAGAAGACGCGGATC	GAGGAGCAGGTTGTGGATGTT	−	−
LOC_Os07g43810	GTGGCTTTGGATTCGTCACA	ATCTTGGCGCGAAATCATCC	−	−
LOC_Os10g39150	GTTCATCAACGCCCTCAAACAA	GAAGCCTGTCAATTGCCGATAC	**	−
LOC_Os11g40150	GTTTGGAGTGGCTGTGGTTA	TGAGCCATGATGTTTCCACC	−	−

+: up-regulated genes
-: down-regulated genes
** null
