# Supplementary material for: Chromatin states responsible for the regulation of differentially expressed genes under 60Co~γ ray radiation in rice
Source: BMC Genomics. 2017 Oct 12;18:778. doi: 10.1186/s12864-017-4172-x (PMC5639768; doi:10.1186/s12864-017-4172-x)
Supplement: Supplementary file 7 — The p-value of Wilxcoxon rank-sum test (one side) for each mark distributed across up-regulated genes between 0 Gy and 50 Gy. (PDF 54 kb) [file 12864_2017_4172_MOESM7_ESM.pdf]

Table S4: The *p*-value of Wilxcoxon rank-sum test (one side) for each mark distributed across up-regulated genes between 0 Gy and 50 Gy

FPKM	H3K4ac	H3K27ac	H4K12ac	H3K4me1	H3K4me3	H3K27me3	H3K36me3
**>50**	0.33760	0.00713	< 2.2e-16	1.63E-13	8.67E-05	0.00023	0.03123
**10~50**	0.002004	0.00024	< 2.2e-16	< 2.2e-16	2.95E-06	< 2.2e-16	2.66E-05
**1~10**	1.84E-11	0.14530	< 2.2e-16	< 2.2e-16	0.42770	5.48E-13	0.01675
**<1**	0.005122	0.13250	0.00087	1.87E-14	0.00076	0.01933	0.20770
