# Supplementary material for: Chromatin states responsible for the regulation of differentially expressed genes under 60Co~γ ray radiation in rice
Source: BMC Genomics. 2017 Oct 12;18:778. doi: 10.1186/s12864-017-4172-x (PMC5639768; doi:10.1186/s12864-017-4172-x)
Supplement: Supplementary file 9 — The p-value of Wilxcoxon rank-sum test (one side) for each mark distributed across down-regulated genes between 0 Gy and 50 Gy. (PDF 54 kb) [file 12864_2017_4172_MOESM9_ESM.pdf]

Table S5: The *p*-value of Wilxcoxon rank-sum test (one side) for each mark distributed across down-regulated genes between 0 Gy and 50 Gy

**FPKM**	**H3K4ac**	**H3K27ac**	**H4K12ac**	**H3K4me1**	**H3K4me3**	**H3K27me3**	**H3K36me3**
**>50**	1.52E-07	2.44E-09	< 2.2e-16	8.10E-06	4.93E-09	< 2.2e-16	0.00046
**10~50**	3.51E-08	3.37E-09	< 2.2e-16	1.57E-12	0.06666	< 2.2e-16	0.14240
**1~10**	9.59E-05	2.08E-06	< 2.2e-16	1.52E-09	7.74E-08	7.67E-12	0.43400
