# Supplementary material for: Chromatin states responsible for the regulation of differentially expressed genes under 60Co~γ ray radiation in rice
Source: BMC Genomics. 2017 Oct 12;18:778. doi: 10.1186/s12864-017-4172-x (PMC5639768; doi:10.1186/s12864-017-4172-x)
Supplement: Supplementary file 11 — Summary of the sequencing mapping of RNA-seq and ChIP-seq data sets. (PDF 37 kb) [file 12864_2017_4172_MOESM11_ESM.pdf]

Table S6: Summary of the sequencing mapping of RNA-seq and ChIP-seq data sets

ID	Treat（Gy）	Time（min）	Total reads (Million)	Mapping ratio (%)	Unique Reads (%)	PE （bp）
RNA-seq	0(CK)-Rep I	0	19,619,836	88.2	81.66	150
RNA-seq	0(CK) -Rep II	0	28,641,205	90.9	84.97	150
RNA-seq	50-Rep I	30	15,793,445	89.0	82.54	150
RNA-seq	50-Rep II	30	15,509,128	88.8	82.14	150
H3K27ac-ChIP-seq	0 (CK)	0	14,626,517	89.48	75.49	50
H3K4ac-ChIP-seq	0(CK)	0	14,808,579	82.07	67.08	50
H4K12ac-ChIP-seq	0(CK)	0	9,056,851	89.87	75.16	50
H3K4me1-ChIP-seq	0(CK)	0	14,054,023	95.50	84.17	50
H3K4me3-ChIP-seq	0(CK)	0	14,981,689	95.79	85.52	50
H3K27me3-ChIP-seq	0(CK)	0	24,493,313	86.29	71.82	50
H3K36me3-ChIP-seq	0(CK)	0	18,915,487	89.64	78.84	50
H3K27ac-ChIP-seq	50	30	14,834,801	96.75	84.08	50
H3K4ac-ChIP-seq	50	30	10,089,525	93.54%	78.61%	50
H4K12ac-ChIP-seq	50	30	11,230,555	91.85%	73.28%	50
H3K4me1-ChIP-seq	50	30	15,727,547	97.19%	89.06%	50
H3K4me3-ChIP-seq	50	30	12,874,673	96.10%	87.66%	50
H3K27me3-ChIP-seq	50	30	13,500,535	92.77%	81.07%	50
H3K36me3-ChIP-seq	50	30	17,360,244	94.76%	85.40%	50

Note: PE represents paired-end sequencing
