# Supplementary material for: Chromatin states responsible for the regulation of differentially expressed genes under 60Co~γ ray radiation in rice
Source: BMC Genomics. 2017 Oct 12;18:778. doi: 10.1186/s12864-017-4172-x (PMC5639768; doi:10.1186/s12864-017-4172-x)
Supplement: Supplementary file 12 — Summary of anti-H4K12ac based ChIP-qPCR assay. (PDF 83 kb) [file 12864_2017_4172_MOESM12_ESM.pdf]

Table S7: Summary of anti-H4K12ac based ChIP-qPCR assay

Locus number	FPKM (0Gy/50Gy)	Forward primer	Reverse primer	ChIP-qPCR (0Gy/50Gy)	*t*-test	ChIP-seq
LOC_Os01g65890	7.1/351.9	TTTCCCCGGGTAAAACTGCC	CCACCTCCATCTCCACGAAC	−	**	−
LOC_Os05g45350	9.7/125.2	GACCTTAGGAGCTGTGCCTATG	GAACACTCACGACGCCAAGAA	−	**	−
LOC_Os06g46950	18.8/197.8	AGGAGGGGTTCGAGGACTAC	CTGTCGAAGGTGATGAGCCC	−	**	−
LOC_Os10g35770	63.8/178.7	AGTTCCTCCGGCTCTGCT	GGCCTGAGGAAGACGGTCT	−	*	−
LOC_Os03g19720	38/123	GAGGACATCGCCGCCATG	GTCGTCGAACCCCACCTT	−	**	−
LOC_Os01g70850	8.2/198.1	GAATTTCGAAGCAGCTGAGC	GACCACCCTGTACCAGCAC	−	**	−
LOC_Os09g27040	1.3/43.8	GTTGTTGATCTTGGCCTGCC	CCCCGTCCATCAAGAAGTCG	+	N	−
LOC_Os06g11240	9.1/34.4	GTGTAGTACAGCACCGCGT	GCTGACGCCGTACAAGATG	−	**	−
LOC_Os01g64120	46.0/120.8	AGCTGCCTTACTCGTGCC	CACGTACCCCTGCTCGAT	−	*	−
LOC_Os01g21160	24.6/68.3	GCTCCTCCTTCTCCTAGTGCT	GCCTACCAATCCATCCAGACC	−	**	−
LOC_Os03g05780	27.6/57.0	AGATGGTGATGACGAGCTTGG	CTCTACCCGGTCTGCTTCTTC	−	**	−
LOC_Os03g56310	19.3/45.4	ATGGGACGGCTAAAACAAAACG	TTGCGGTTCTCTGGTGATTCTT	−	*	−
LOC_Os01g50622	147.8/1.1	CTGTTTGTTGCAGTTACGGCTC	AATTACCAGCCTCCATCACGC	−	**	−
LOC_Os03g01750	122.1/42.9	CTGTTCGTCACTGCTGGATTTC	AATGGAATGGAAGAGGGGTCAC	−	*	−
LOC_Os06g50080	101.41/23.9	CAGGAGCAGATGGACATGGAC	GGAGTACACAACACTAGCTGGT	−	*	−
LOC_Os09g32250	31/15	GCTCAGGGCGAAGATCTCC	CCATCGAACCCCAAATGAAGC	−	*	−
LOC_Os10g38910	93.7/3.1	GAGAGGTTGGTTGTGGAGGT	AAACCAGCGAAACCACCATC	−	**	−
LOC_Os08g32930	94.9/5.2	CTCAGATTTAGCCGCCGTCG	GTAACGATGCTCCCTCCCCG	−	**	−
LOC_Os06g26234	29/11.3	CGGTTTCCCCTCTGCTAAATT	CAGGCAAATCTGTCAGCAATAT	−	**	−
LOC_Os03g48040	143.1/64.6	CGTCGTCCCACAGGTTAATCA	GCACGAATCAGACAAAGCACA	−	**	−
LOC_Os07g30670	249.4/14.5	AGCACAAACAGCAGAGGAGG	AGCGAGGAATCACTGAATCGG	−	**	−
LOC_Os08g02400	196.5/94.6	CTTCTTCGCGGCCTTCATGAT	TTCCATTCCCTTTTCACGGTGA	−	**	−

-: decrease in H4K12ac post IR; +: more enriched H4K12ac post-IR
**: *p*<0.01 in *t*-test; *: *p*<0.05 in *t*-test; N:not significant in *t*-test
